# Sugarcane Molasses Polyphenol Extract Attenuates Alcohol-Induced Chronic Liver Damage via Antioxidant, Anti-Inflammatory, and CYP2E1/Keap1/NF-κB Pathway Modulation

**DOI:** 10.3390/nu17091589

**Published:** 2025-05-05

**Authors:** Min Wang, Lin Zhao, Yumei Wang, Chengfeng Zhang, He Li

**Affiliations:** 1 Key Laboratory of Geriatric Nutrition and Health, Beijing Technology and Business University, Beijing 100048, China; balance0831@163.com (M.W.); sdtzzhaolin@163.com (L.Z.); wym110809@126.com (Y.W.); 2 The Product Makers Co., Ltd., Shanghai 200444, China; eric.zhang@theproductmakers.cn

**Keywords:** natural active substances, sugarcane polyphenols, chronic diseases, alcoholic liver damage, oxidative stress, inflammation, hepatoprotective activity

## Abstract

Background/Objective: The prevention and treatment of alcoholic liver disease (ALD) urgently require safe and effective nutritional intervention strategies. Polyphenol extracts from sugarcane molasses (SP) show antioxidant and anti-inflammatory potential, yet their protective effects against ALD have not been elucidated. This study explored the therapeutic potential of SP in alcohol-induced chronic liver damage. Methods: A graded alcohol concentration-induced liver damage model was established in C57BL/6J mice to systematically evaluate SP’s regulatory effects on liver function markers, lipid metabolism, oxidative stress indicators, inflammatory factors, and related molecular mechanisms through a 10-week nutritional intervention. Results: The results demonstrated that SP intervention significantly inhibited the liver index, alanine aminotransferase and aspartate aminotransferase activities, and triglyceride and total cholesterol accumulation in mice. SP enhanced antioxidant enzyme activities in a dose-dependent manner, with the high-dose group increasing catalase activity by 161.19% and superoxide dismutase activity by 22.97%. Furthermore, SP significantly reduced the levels of pro-inflammatory cytokines, including interleukin-1β, interleukin-6, and tumor necrosis factor-α, thereby alleviating hepatic inflammatory infiltration. Mechanistic studies revealed that SP effectively mitigated alcohol-induced oxidative stress and inflammatory injury by inhibiting cytochrome P450 2E1 overexpression, regulating the Kelch-like ECH-associated protein 1 signaling pathway, and suppressing nuclear factor-kappa B pathway activation. Conclusions: The findings reveal that SP mitigates ALD via synergistic antioxidant and anti-inflammatory mechanisms, providing a novel strategy for high-value utilization of sugarcane molasses byproducts in agricultural industries. Future studies should focus on the contribution of the different phenolics in SP and validate their specific hepatoprotective mechanisms.

## 1. Introduction

Chronic diseases have emerged as a major global public health threat, among which alcoholic liver disease (ALD) exhibits characteristic progressive pathological stages: initiating as alcoholic fatty liver, advancing to steatohepatitis and fibrosis, and potentially culminating in hepatocellular carcinoma [1]. The progression of ALD is a cumulative and time-dependent process influenced by multiple factors, rather than a simple linear consequence of alcohol consumption [2]. Ethanol metabolism in the liver primarily occurs through alcohol dehydrogenase (ADH) and aldehyde dehydrogenase (ALDH), generating acetaldehyde and acetate while producing reactive oxygen species (ROS) [3,4]. Cytochrome P450 2E1 (CYP2E1) has been shown to be an ethanol-metabolizing enzyme whose chronic overactivation contributes to ROS and acetaldehyde accumulation [5]. The resulting oxidative stress represents a central pathogenic mechanism in ALD, directly inducing hepatocyte damage through lipid peroxidation, protein damage, and nucleic acid modification, while simultaneously triggering inflammatory responses [6,7,8]. These inflammatory mediators subsequently aggravate mitochondrial dysfunction, establishing a vicious cycle of mutually reinforced oxidative stress and inflammation that ultimately worsens hepatic injury [1]. Under conditions of oxidative stress, an imbalance occurs in both the Kelch-like ECH-associated protein 1 (Keap1) pathway and the nuclear factor-κB (NF-κB) signaling pathway [9,10]. Disruption of the Keap1 pathway compromises redox homeostasis, while pro-inflammatory cytokines, including interleukin-1β (IL-1β), interleukin-6 (IL-6), and tumor necrosis factor-α (TNF-α), are transcriptional targets of NF-κB and play central roles in amplifying hepatic inflammation [6,11]. Consequently, targeted modulation of these oxidative stress and inflammatory pathways has been identified as a crucial therapeutic strategy for ALD management [12]. Current pharmacological interventions face limitations, including high costs and suboptimal efficacy. In contrast, dietary polyphenols have emerged as a research focus for chronic disease prevention and treatment due to their multi-target regulatory capacity, such as antioxidant, anti-inflammatory, lipid metabolism modulation, gut microbiota composition improvement, and anti-apoptotic activities, demonstrating unique advantages in ALD intervention [13,14,15,16]. For example, hawk tea flavonoids (HTFs) ameliorated alcohol-induced liver damage by modulating gut microbiota composition and regulating the nuclear factor erythroid 2-related factor 2 (Nrf2)/NF-κB signaling pathways [12]. Mulberry leaf polyphenols improved abnormal lipid metabolism and apoptosis signaling in mice, among other dietary polyphenols reported to exert hepatoprotective effects [17,18]. However, these studies primarily focused on high-cost plant extracts, making the exploration of economical and sustainable novel bioactive sources an urgent priority.

Sugarcane molasses, a primary byproduct of the sugar industry with an annual production of 34 million tons, is rich in polyphenols such as chlorogenic acid (CA) and caffeic acid, which show potential for intervention in chronic diseases [19,20]. However, its current applications remain largely confined to low-value uses such as animal feed and fermentation substrates, with markedly underdeveloped high-value utilization [21]. Our previous studies have demonstrated that sugarcane molasses polyphenol extract (SP) exhibits significant antioxidant and anti-inflammatory activities [22,23]. In UV-B-induced skin photoaging mouse models, SP effectively attenuated oxidative stress and inflammation by enhancing the activities of antioxidant enzymes including catalase (CAT) and superoxide dismutase (SOD), while simultaneously suppressing pro-inflammatory cytokines such as IL-1β, IL-6, and TNF-α [22]. Furthermore, SP protected against advanced glycation end products (AGEs)-induced cellular injury by decreasing ROS levels and suppressing activation of the phosphatidylinositol 3-kinase/protein kinase B (PI3K/Akt) pathway [23]. Therefore, the conversion of sugarcane molasses into functional food ingredients for ALD intervention represents a promising strategy to achieve high-value resource utilization and chronic disease management simultaneously. Although previous studies have elucidated certain mechanisms of polyphenols in ALD intervention, the hepatoprotective effects of SP and its molecular mechanisms in alcohol-induced liver damage remain unexplored [14,24].

This study adopted a chronic alcohol-feeding mouse model to comprehensively evaluate the hepatoprotective effects of SP by assessing liver function biomarkers, oxidative stress parameters, and inflammatory markers. The findings demonstrate that SP mitigates alcohol-induced liver damage through coordinated regulation of multiple molecular pathways. By systematically assessing SP’s modulatory effects on chronic alcohol-induced liver damage, this research not only provides a novel agricultural byproduct-based nutritional intervention strategy for the dietary prevention of ALD but also opens innovative avenues for the high-value utilization of sugarcane molasses.

## 2. Materials and Methods

### 2.1. Materials

The SP (Phytolin^®^) was supplied by The Product Makers Co. Ltd. (Shanghai, China). The SP contained an 18 mg gallic acid equivalent/mL of total polyphenols. CA was the most abundant phenolic substance, with a content of 146.38 μg/g, representing the characteristic component of SP. The other major phenolic compounds in the SP included p-coumaric acid, vanillin, butyric acid, and caffeic acid at concentrations of 20.69 μg/g, 18.97 μg/g, 1.08 μg/g, and 0.96 μg/g [22].

The CA was purchased from Shanghai Yuanye Biotechnology Co. Ltd. (Shanghai, China) while the anhydrous ethanol was obtained from Shanghai McLean Biochemical Co. Ltd. (Shanghai, China). The alanine aminotransferase (ALT), aspartate aminotransferase (AST), triglyceride (TG), total cholesterol (TC), CAT, SOD, and malondialdehyde (MDA) kits were purchased from Nanjing Jiancheng Bioengineering Institute (Nanjing, China), while the IL-1β, IL-6, and TNF-α were obtained from Hangzhou Lianke Biotechnology Co. Ltd. (Hangzhou, China). The radioimmunoprecipitation assay (strong RIPA lysate), phenylmethylsulfonyl fluoride (PMSF, 100 mM), phosphatase inhibitor mixture A (50×), Keap1, CYP2E1, phospho-IKKα/β (Ser176/180) rabbit polyclonal antibody, rabbit monoclonal antibody against IKKα/β, GAPDH, HRP-labelled goat anti-rabbit IgG (H+L), ultrasensitive ECL chemiluminescence kit, and polyvinylidene difluoride (PVDF) membrane were supplied by Beyotime Biotechnology Co Ltd. (Shanghai, China). The heme oxygenase 1 (HO-1), rabbit monoclonal antibody to IκBα, phospho-IκBα (Ser32/Ser36), NF-κB p65, and phospho-NF-κB p65 (Ser536) rabbit polyclonal antibodies were purchased from Chengdu Zhengneng Biotechnology Limited (Chengdu, China) while the 10× Tris-buffered saline with Tween-20 (TBST) buffer was acquired from Wuhan Service Biotechnology Co. Ltd. (Wuhan, China).

### 2.2. Animal Tests

Five-week-old male SPF-grade C57BL/6J mice weighing 19.52 ± 0.8 g were purchased from Beijing Huafukang Biotechnology Co. (Beijing, China) and housed at 25 ± 2 °C, 60% relative humidity, with a 12 h light–dark cycle and free access to food and water. The experiment was conducted at Beijing Huayuan Time Technology Co. Ltd., (Beijing, China) and the test protocol was approved by its Animal Research Ethics Committee (Animal Ethics No. HYSD2024-02).

**Figure 1:**
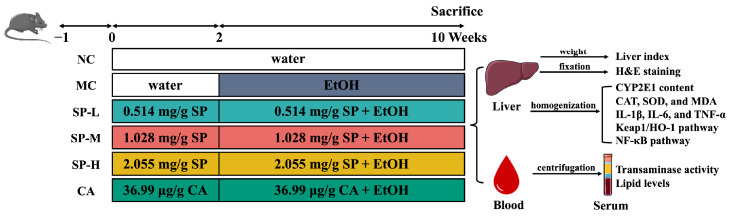
The animal test groups and procedures.

After adaptation feeding, the mice were randomly divided into six groups (*n* = 6): the Normal Control (NC) group, the Model Control (MC) group, the SP Low-Dose (SP-L) group, the SP Medium-Dose (SP-M) group, the SP High-Dose (SP-H) group, and the CA group. Pregavage lasted for two weeks. During this period, the NC and MC groups were gavaged with ultrapure water daily in the morning, the SP-L, SP-M, and SP-H groups were gavaged with 0.514 mg/g, 1.028 mg/g, and 2.055 mg/g of BW SP, and the CA group was gavaged with 36.99 μg/g of BW CA. The SP gavage quantity for the SP-H group was converted to the recommended daily intake (≤10 g/60 Kg) for humans [22]. The SP-H group had the same polyphenol content as the CA group. The pregavage period was followed by gradient alcohol gavage to simulate human drinking [25]. This process utilized a gradient modeling method delineated by Xia et al., with some modifications [26,27]. The experimental flow is shown in Figure 1. Then, 2 h after the end of gavage in the morning, the NC group was gavaged with ultrapure water at 10 mL/kg BW per day. In contrast, the MC group and the nutritional intervention groups with different SP and CA concentrations were gavaged with 30% (*v*/*v*) ethanol for one week, 40% (*v*/*v*) ethanol for two weeks, and 50% (*v*/*v*) ethanol for five weeks. After a 12 h fasting period, the mice were anesthetized and subsequently euthanized. Whole blood was collected and maintained at room temperature for 20 min, followed by centrifugation at 4 °C (4000 r/min for 15 min) to obtain serum. The serum was then aliquoted, flash-frozen in liquid nitrogen, and stored at −80 °C for subsequent biochemical analysis. The mouse livers were rinsed in PBS and their weights were recorded. Some of the liver tissue was immediately fixed with 4% paraformaldehyde for histological analysis, while the remaining tissue was frozen in liquid nitrogen and stored at −80 °C for further analysis.

### 2.3. Liver Index Analysis

The liver index was calculated using the following formula: mouse liver index (%) = (liver weight/body weight) × 100% [17].

### 2.4. Biochemical Analysis

Serum ALT, AST, TG, and TC levels were determined using kits obtained from Nanjing Jiancheng (Nanjing, China). The serum was thawed on ice, tested in 96-well plates, and measured using an enzyme reader. CAT, SOD, and MDA kits (Nanjing Jiancheng, Nanjing, China) were used to measure the corresponding activity levels. The tissue homogenization method was adapted from Wang et al., with modifications [22]. Saline was added to an appropriate quantity of liver tissue, and the mixture was homogenized at a 1:9 ratio on ice to maintain a low temperature. After centrifugation at 12,000 r/min for 20 min at 4 °C, the supernatant was collected for measurement. The assay was performed according to the kit instructions.

### 2.5. Histological Analysis

The experimental protocol was adapted from Xu et al., with appropriate modifications [12]. Pre-fixed liver tissues were embedded in paraffin, sectioned into 5 μm slices for hematoxylin and eosin (H&E) staining, dehydrated, and sealed with neutral gum. The samples were examined under a microscope to assess the impact of different treatments on liver histopathology.

### 2.6. Enzyme-Linked Immunosorbent Assay (ELISA)

Liver tissues were homogenized as described in Section 2.5, and protein concentrations were measured. IL-1β, IL-6, and TNF-α inflammatory cytokine levels were determined using ELISA kits (Hangzhou Lianke, Hangzhou, China), and the results were expressed as protein concentrations [22]. Assays were performed following the kit instructions.

### 2.7. Western Blot

The experimental methods were performed according to Wang et al., with modifications [23]. An appropriate amount of mouse liver tissue was collected, mixed with RIPA lysate containing PMSF and a phosphatase inhibitor, homogenized using a handheld grinder, and incubated on ice for 30 min, followed by centrifugation at 12,000 r/min and 4 °C for 20 min. The supernatant was collected and analyzed using a bicinchoninic acid (BCA) assay, after which the protein concentration was normalized. Then, protein loading buffer was added, followed by denaturing at 100 °C in a metal bath to prepare the test samples. The samples from the different treatment groups were separated using sodium dodecyl sulfate–polyacrylamide gel electrophoresis (SDS-PAGE). Afterward, the proteins on the gel were transferred onto PVDF membranes via electrotransfer. The PVDF membranes were blocked with 5% skimmed milk or bovine serum albumin for 1 h, washed three times with 1× TBST for 5 min each, and incubated overnight at 4 °C with specific primary antibodies (GAPDH at a dilution of 1:10,000, and other antibodies at 1:1000). Next, the membranes were rewashed with TBST, incubated with the secondary antibody (dilution 1:2000) at 25 °C for 2 h, washed three times with 1× TBST, immersed in an ECL luminescent solution for 10 s, and imaged using a gel imaging analysis system. ImageJ (version 2.14.0) was used to quantitatively analyze the grayscale values to assess the effect of different treatments on target protein expression.

### 2.8. Statistical Analysis

The experimental data were expressed as mean ± standard deviation and statistically analyzed using SPSS Statistics (version 26.0; IBM Corp., Armonk, NY, USA). Group differences were analyzed by one-way ANOVA followed by Duncan’s test (for homogeneous variance, Levene’s *p* > 0.05) or Games–Howell test (for heterogeneous variance, Levene’s *p* ≤ 0.05), with statistical significance set at *p* < 0.05. The Western blot analysis was repeated three times while six replicates were obtained for all other experimental indices.

## 3. Results

### 3.1. The Effect of SP Nutritional Intervention on the Body Weights and Liver Index of the ALD Mice

**Figure 2:**
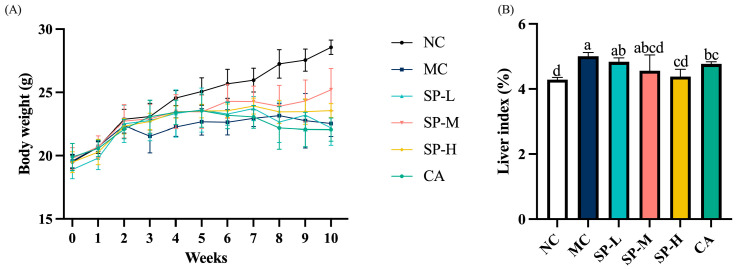
The effect of SP nutritional intervention on the body weights and liver index of the ALD mice. (**A**) The average body weights of the mice. (**B**) The liver index of the mice. Note: Different lowercase letters denote significant differences among the groups (*p* < 0.05).

The body weights of the ALD mice were recorded to investigate the effect of SP nutritional intervention. The mice in the NC group exhibited higher body weights (*p* < 0.05), whereas those in the MC group showed no significant differences compared to the nutritional intervention groups receiving different SP and CA doses (Figure 2A).

The MC group exhibited a higher liver index than the NC group (*p* < 0.05), indicating that EtOH induced an abnormally high liver index in the ALD mice (Figure 2B). A higher SP dose gradually decreased the liver index of the mice in the different SP intervention groups to 3.44%, 8.82%, and 12.37%, respectively, compared to the MC group, while that of the SP-H group declined substantially (*p* < 0.05). The CA group also showed a significant decrease in liver index of 4.72% compared with the MC group (*p* < 0.05). The SP-H and CA groups showed a similar ability to prevent a rise in the liver index in the mice. The results revealed that although SP did not significantly affect body weight, high-dose nutritional intervention effectively suppressed alcohol-induced liver index abnormalities.

### 3.2. The Effect of SP Nutritional Intervention on the Serum Transaminase and Lipid Levels in the ALD Mice

**Figure 3:**
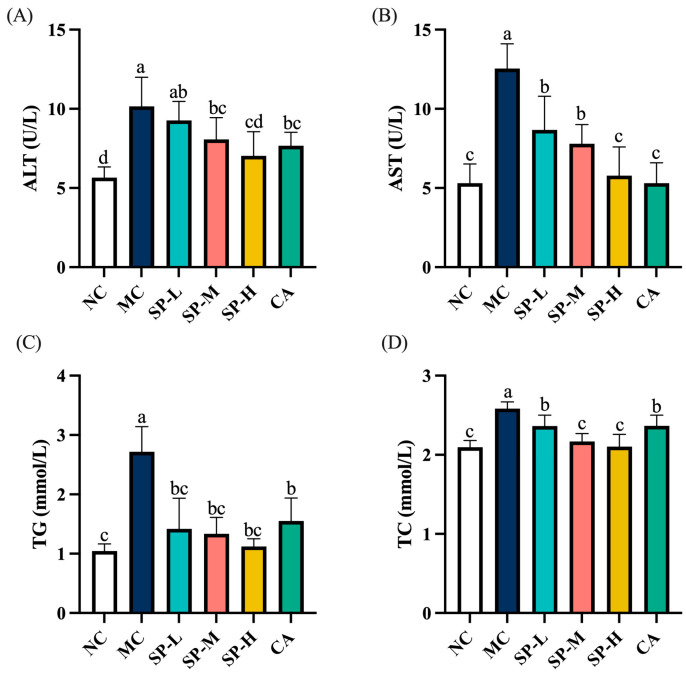
The effect of SP nutritional intervention on serum levels of (**A**) ALT, (**B**) AST, (**C**) TG, and (**D**) TC in ALD mice. Note: Different lowercase letters denote significant differences among the groups (*p* < 0.05).

The serum transaminase and lipid levels were measured to investigate the protective effect of SP nutritional intervention on the liver of ALD mice. As shown in Figure 3A,B, the ALT and AST levels in the MC group significantly exceeded the values in the NC group by 1.79-fold and 2.37-fold (*p* < 0.05), respectively. SP nutritional intervention gradually decreased ALT and AST activity in a dose-dependent manner, with high doses yielding levels similar to those in the NC group. The CA group displayed significantly lower transaminase activity than the MC group (*p* < 0.05). SP-H treatment exhibited a similar effect to CA in reducing serum transaminase activity in the ALD mice. The results indicated that SP nutritional intervention effectively reduced the EtOH-induced elevation in serum transaminase activity in the ALD mice.

As shown in Figure 3C,D, the TG and TC levels were 2.60-fold and 1.23-fold higher in the MC group than in the NC group (*p* < 0.05). The TG and TC levels decreased significantly after treatment with different SP doses (*p* < 0.05). A low SP dose reduced the TG level to a value similar to that of the NC group. At the medium dose of SP nutritional intervention, the TC level did not differ significantly from that of the NC group. In contrast, the CA group exhibited significantly elevated TG and TC levels (*p* < 0.05). The ability of a high SP dose to modulate the TC level surpassed that of CA. The results indicated that SP attenuated EtOH-induced hepatocellular damage by lowering serum ALT and AST levels and improving lipid metabolism while reducing abnormal TG and TC accumulation in the serum. These findings confirmed the multifaceted protective effect of SP nutritional intervention in mitigating ALD.

### 3.3. The Effect of SP Nutritional Intervention on the Liver CYP2E1 Content and Oxidative Stress in the ALD Mice

**Figure 4:**
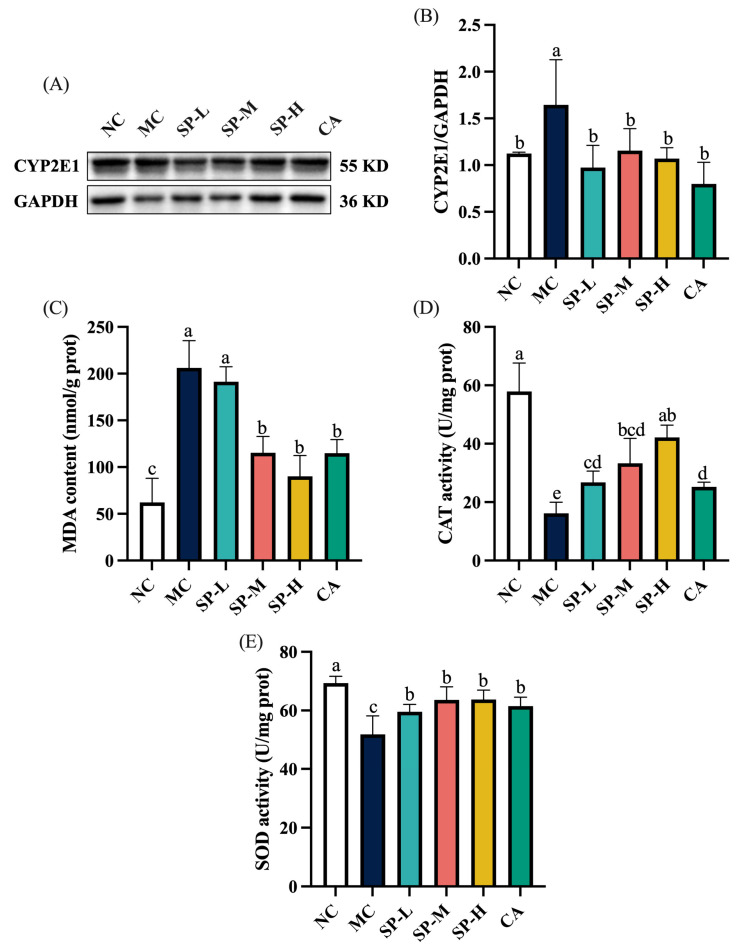
The effect of SP nutritional intervention on the liver CYP2E1 content and oxidative stress in the ALD mice. (**A**) The representative CYP2E1 protein blot. (**B**) The CYP2E1 protein content. (**C**) MDA content. (**D**) CAT activity. (**E**) SOD activity. Note: Different lowercase letters denote significant differences among the groups (*p* < 0.05).

Prolonged EtOH intake activates the alternative alcohol oxidation pathway mediated by CYP2E1, which results in CYP2E1 overexpression [28]. As shown in Figure 4A,B, the MC group displayed a significantly elevated CYP2E1 protein level, which was 1.46-fold higher than in the NC group (*p* < 0.05). Different SP nutritional intervention doses significantly inhibited excessive CYP2E1 upregulation (*p* < 0.05). The CYP2E1 expression in the SP-L, SP-M, and SP-H groups did not differ substantially from the NC group, despite the absence of dose dependence, while low-dose SP nutritional intervention effectively downregulated CYP2E1 overactivation (*p* < 0.05). The CA group also exhibited significantly lower CYP2E1 protein expression levels than the MC group (*p* < 0.05). The SP-H and CA groups displayed comparable effects in suppressing CYP2E1 expression. The results demonstrated that SP nutritional intervention inhibited CYP2E1 overexpression in the liver of the ALD mice, consequently influencing alcohol metabolism and mitigating oxidative stress damage.

Alcohol metabolism generates reactive ROS, which cause oxidative stress and tissue damage. As illustrated in Figure 4C, the MDA level increased substantially in the MC group and was 3.31-fold higher than in the NC group (*p* < 0.05). Nutritional intervention with different SP doses gradually reduced the MDA level in a dose-dependent manner. Moreover, the CA group exhibited significantly lower MDA levels than the MC group (*p* < 0.05), while high-dose SP intervention displayed similar MDA reduction efficacy to CA treatment. As shown in Figure 4D,E, the CAT and SOD activity in the liver of the mice in the MC group decreased significantly to 16.13 ± 3.85 U/mg prot and 51.83 ± 6.32 U/mg prot, respectively, compared with the NC group (*p* < 0.05). SP nutritional intervention enhanced the CAT and SOD activity in a dose-dependent manner. Compared with the MC group, the CAT activity in the SP treatment groups increased by 66.02%, 106.39%, and 161.19%, while SOD levels rose by 14.92%, 22.77%, and 22.97%, respectively. A low SP dose significantly increased the CAT and SOD activity (*p* < 0.05). Furthermore, the CA group also exhibited substantially higher CAT and SOD levels, which increased by 56.35% and 18.69% (*p* < 0.05), respectively. Additionally, the SP-H group displayed considerably higher CAT activity than the CA group. The results confirmed that SP nutritional intervention counteracted the oxidative stress induced by chronic alcohol exposure in the ALD mice in a dose-dependent manner, as evidenced by a significant increase in the CAT and SOD activity, and a considerable reduction in the MDA levels.

### 3.4. The Effect of SP Nutritional Intervention on the Liver Pathology and Inflammatory Factors in the ALD Mice

**Figure 5:**
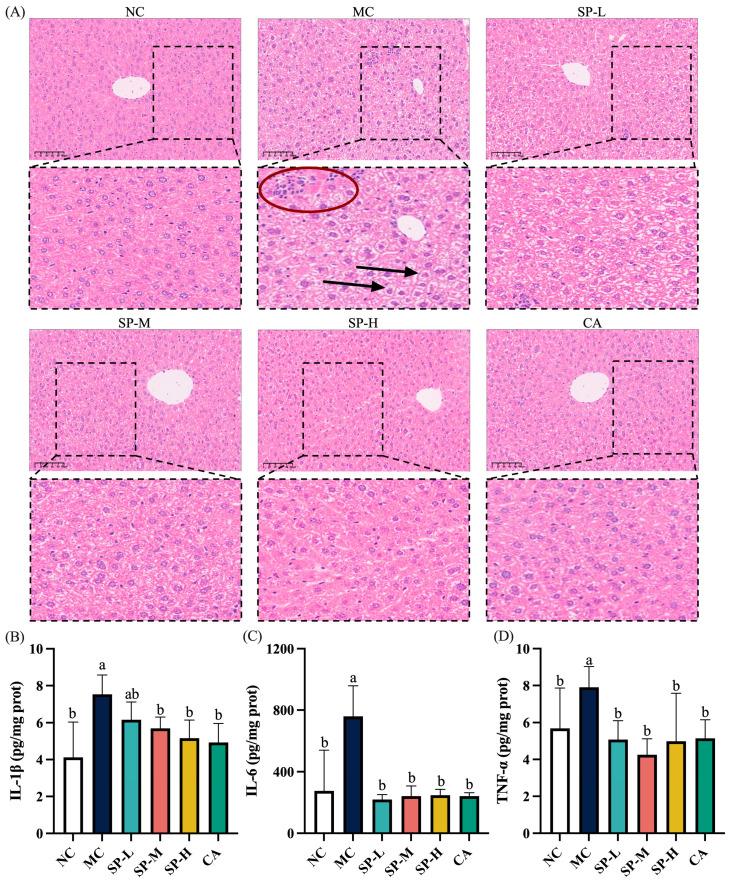
The effect of SP nutritional intervention on the liver pathology and inflammatory factors in the ALD mice. (**A**) Pathological sections of the liver. (**B**) IL-1β. (**C**) IL-6. (**D**) TNF-α. Scale bar = 100 μm. The black arrows denote hepatocyte edema, while the red ellipse represents inflammatory cell infiltration. Note: Different lowercase letters denote significant differences among the groups (*p* < 0.05).

H&E staining was used to examine the histopathological changes in the liver of the ALD mice to determine the impact of SP nutritional intervention. The liver tissue of the mice in the NC group was intact with normal hepatocyte structures and a uniformly stained cytoplasm with clear nuclei (Figure 5A). The MC group presented typical ALD pathological features, including hepatocyte edema (black arrows) and inflammatory cell infiltration (red ellipse). In the different doses of the SP nutritional intervention groups, the liver tissue morphology of the ALD mice gradually improved, and the hepatocyte structure became increasingly normal in a dose-dependent manner. Among them, the SP-H group showed results most similar to those of the NC group. Compared with the MC group, the CA group also exhibited some improvement in liver tissue structure and hepatocyte morphology, with effects similar to those of the SP-M group. The results indicated that SP nutritional intervention improved liver tissue damage induced by EtOH in ALD mice, maintained normal hepatocyte structure, and alleviated inflammatory infiltration.

Excessive alcohol consumption increased inflammatory cytokine expression in the liver of the mice [29,30]. The levels of IL-1β, IL-6, and TNF-α were measured to investigate the impact of SP nutritional intervention on liver inflammation in ALD mice. As shown in Figure 5B–D, the IL-1β, IL-6, and TNF-α increased significantly in the MC group (*p* < 0.05). These values were 1.83-fold, 2.76-fold, and 1.39-fold, respectively, higher than in the NC group. Conversely, SP nutritional intervention significantly reduced the levels of IL-1β, IL-6, and TNF-α, with the SP-M and CA groups exhibiting the more pronounced effects (*p* < 0.05). The SP-H and CA groups exhibited similarly reduced inflammatory factor levels. Therefore, SP nutritional intervention substantially inhibited the IL-1β, IL-6, and TNF-α levels in the liver of the ALD mice, consequently mitigating liver inflammation.

### 3.5. The Effect of SP Nutritional Intervention on the Keap1/HO-1 Signaling Pathway in the ALD Mice

**Figure 6:**
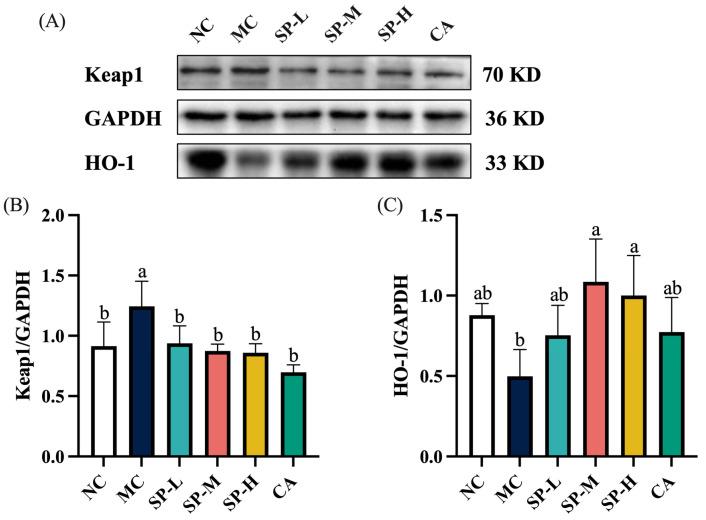
The effect of SP nutritional intervention on the Keap1/HO-1 signaling pathway in the ALD mice. (**A**) The representative Keap1 and HO-1 protein blots. (**B**) The Keap1 content. (**C**) The HO-1 protein. Note: Different lowercase letters denote significant differences among the groups (*p* < 0.05).

The activation of the Keap1/HO-1 signaling pathway is crucial during oxidative stress, necessitating the assessment of the Keap1 levels and the downstream protein HO-1. As shown in Figure 6A,B, the Keap1 protein was significantly upregulated, being 1.63-fold higher in the MC group than in the NC group (*p* < 0.05). SP nutritional intervention significantly inhibited Keap1 protein expression in a dose-dependent manner, while the low-dose SP group displayed 18.61% lower Keap1 protein levels (*p* < 0.05). Similarly, the CA nutritional intervention significantly downregulated EtOH-induced Keap1 protein overexpression (*p* < 0.05). HO-1 protein expression was 43.30% lower in the MC group compared to the NC group (Figure 6A,C). SP nutritional intervention substantially upregulated HO-1 protein expression by 2.18-fold and 2.01-fold in the SP-M and SP-H groups, respectively, compared to the MC group. However, the CA group did not exhibit significant upregulation of HO-1 protein expression, which was similar to the SP-H group. SP nutritional intervention regulated the expression of the Keap1 signaling pathway and the HO-1 downstream protein, thereby mitigating EtOH-induced oxidative stress and oxidative damage in ALD mice.

### 3.6. The Effect of SP Nutritional Intervention on the NF-κB Signaling Pathway in the ALD Mice

**Figure 7:**
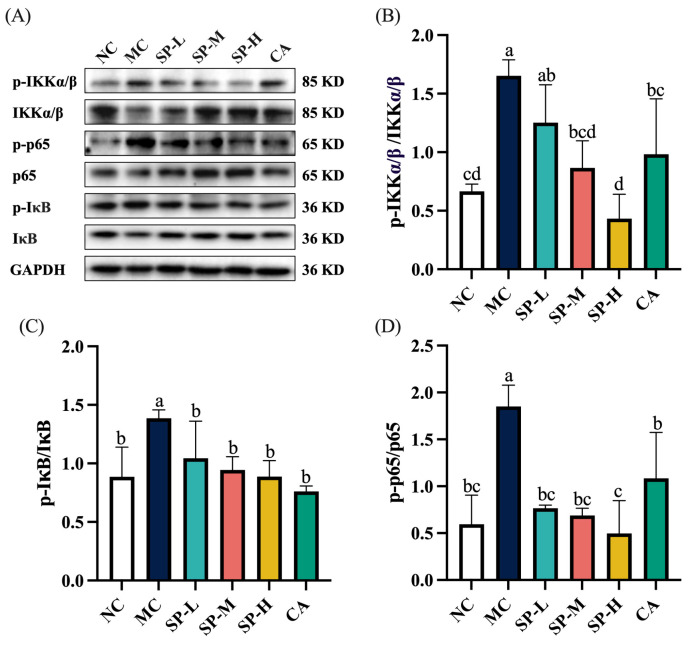
The effect of SP nutritional intervention on the NF-κB signaling pathway in the ALD mice. (**A**) The representative blot of the NF-κB signaling pathway, (**B**) p-IKK, (**C**) p-IκB, and (**D**) p-p65 protein content. Note: Different lowercase letters denote significant differences among the groups (*p* < 0.05).

The NF-κB signaling pathway has attracted considerable attention in ALD research [24]. To examine the effect of SP nutritional intervention on the NF-κB signaling pathway in the ALD mice, the expression of the NF-κB and upstream-related proteins was analyzed using a protein blotting assay. As shown in Figure 7, the MC group displayed significantly elevated p-IKK, p-IκB, and p-p65 protein expression levels, which were 2.48-fold, 1.56-fold, and 3.11-fold higher, respectively, than in the NC group (*p* < 0.05). SP nutritional intervention downregulated the excessive phosphorylation of IKK in a dose-dependent manner, exhibiting a more pronounced suppressive effect in the SP-M group compared to the MC group (*p* < 0.05). A low SP dose decreased the p-IκB and p-p65 proteins to levels comparable to the NC group. CA nutritional intervention significantly inhibited IKK, IκB, and p65 protein phosphorylation (*p* < 0.05). The SP-H group demonstrated a more pronounced inhibitory effect on IKK and p65 phosphorylation than the CA group. The findings indicated that SP nutritional intervention regulated the NF-κB signaling pathway in the ALD mice, while suppressing the upstream p-IKK and p-IκB levels, further preventing p-p65 protein activation.

## 4. Discussion

Alcohol-induced liver damage has become a significant public health concern [31]. In the liver, over 90% of ingested alcohol is metabolized, generating ROS and intermediate metabolites that contribute to oxidative stress and inflammatory injury [6]. Exploring natural bioactive compounds for preventing and alleviating alcoholic liver damage represents a potential nutritional intervention strategy. In this study, we systematically investigated the hepatoprotective effects and molecular mechanisms of SP and its characteristic component CA against chronic alcohol-induced liver damage. Our findings demonstrated that SP exerted significant hepatoprotective effects by modulating multiple targets, including inhibition of CYP2E1 expression, modulation of the Keap1/HO-1 pathway, and suppression of the NF-κB-mediated inflammatory response. These results provide novel evidence that SP protects against alcohol-induced liver damage through a multi-target regulatory network.

### 4.1. SP Regulation of the Transaminase Activity and Lipid Accumulation

The liver index partially reflects the occurrence of liver damage. ALT and AST are typically found in hepatocytes and are released during liver damage, causing elevated levels [32]. Therefore, effectively reducing the liver index and transaminase activity can protect against EtOH-induced liver damage. Previous research has shown that red raspberry polyphenol (RRP) nutritional supplementation reduces the liver index and alleviates EtOH-induced liver damage by suppressing ALT and AST activity in mice [25]. This study revealed that eight weeks of EtOH intake elevated the liver index, ALT, and AST of the mice in the MC group, which was consistent with previous findings. The results indicated that SP nutritional intervention mitigated the elevation in the liver index and the serum ALT and AST release caused by chronic alcohol exposure, consequently providing liver protection. CA nutritional intervention also suppressed the ALT and AST levels, with no significant differences from the SP-H group. This suggested that the other phenolic substances in SP displayed a similar effect to CA in regulating the ALT and AST levels. Previous studies have shown that CA protects against alcoholic liver damage in mice [32]. Kim et al. demonstrated that CA attenuated hepatic oxidative stress in ALD mice by scavenging ROS and protecting hepatocytes from deoxyribonucleic acid (DNA) damage and inflammatory responses, consequently inhibiting hepatic steatosis, hepatocyte apoptosis, and the development of hepatic fibrosis [33]. In the current study, CA was a characteristic component of SP, while the SP-H and CA groups displayed comparable polyphenol content. Selecting CA as a control group allowed the establishment of a known effective reference and the utilization of the quantitative relationship between the SP-H and CA groups to determine whether the impact of SP was primarily attributable to CA or if other phenolics in SP contributed synergistically.

Alcoholic fatty liver disease represents the earliest stage of ALD [34]. Alcohol metabolism in hepatocytes increased the ratio of reduced nicotinamide adenine dinucleotide/oxygenated nicotinamide adenine dinucleotide (NADH/NAD^+^), which enhanced lipid synthesis and inhibited free fatty acid β-oxidation, resulting in TG accumulation in the liver [1,35]. The study found that TG and TC levels were significantly increased in the MC group, suggesting that chronic alcohol exposure induced lipid metabolism disorders and accumulation in the ALD mice. SP nutritional intervention reversed EtOH-induced TG and TC accumulation, mitigating further liver damage. The SP-H group showed more significantly reduced TC accumulation than the CA group, presumably due to the synergistic effect of other phenolics in SP. The reduction in TG and TC accumulation by SP may be related to its influence on alcohol metabolism and antioxidant capacity, consequently affecting lipid synthesis and transport.

### 4.2. SP Modulation of CYP2E1-Mediated Alcohol Metabolism and Oxidative Stress

The prolonged, excessive EtOH ingestion activated the CYP2E1 metabolic pathway, resulting in significant ROS production mediated by CYP2E1 [36,37]. The excessive accumulation of ROS triggered oxidative stress, resulting in liver damage and lipid peroxidation [5]. Therefore, the effective regulation of CYP2E1-mediated alcohol metabolism was considered a reliable approach for alleviating EtOH-induced liver damage. Wang et al. reported that E Se tea polyphenol extract significantly reduced hepatic CYP2E1 expression and prevented severe oxidative stress [38]. The results of the current study demonstrated substantially elevated EtOH-induced CYP2E1 protein expression levels in the MC group, which were suppressed by SP nutritional intervention. CA also significantly downregulated CYP2E1 expression, showing no substantial difference from the SP-H group, suggesting that other phenolics in SP contributed to the decline in the CYP2E1 expression. Previous research has shown that the planar structure of the 2,3-double bond in the C ring of flavonoids can inhibit CYP2E1 [39,40]. Therefore, it was hypothesized that SP, which was rich in phenolics, including Homoorientin and Vitexin, possessed such a structure, which helped to downregulate CYP2E1 expression.

Long-term alcohol consumption induces oxidative stress in the body, which responds by activating enzymatic antioxidant defenses to resist oxidative damage [41]. Du et al. demonstrated that the polyphenol extract derived from fu brick tea promoted SOD and glutathione peroxidase (GSH-Px) activity and reduced MDA levels to alleviate EtOH-induced alcoholic liver damage [42]. The present study indicated that EtOH accelerated CAT and SOD activity decline and promoted MDA accumulation, suggesting that the mice in the MC group displayed impaired antioxidant systems. SP nutritional intervention significantly enhanced CAT and SOD activity while reducing MDA levels, effectively mitigating EtOH-induced oxidative stress and protecting the liver of ALD mice. Compared with the CA group, the SP-H group demonstrated superior efficacy in enhancing CAT activity. This enhanced effect was attributed to the synergistic interactions among diverse polyphenolic constituents present in SP, as opposed to the single-component CA treatment. Combined with previous findings, SP effectively inhibited CYP2E1 protein expression and attenuated CYP2E1-mediated alcohol metabolism, consequently reducing ROS generation. SP also displayed antioxidant properties, enhancing CAT and SOD activity and reducing MDA generation. Experimental results demonstrated that SP significantly attenuated ethanol-induced hepatic oxidative stress and lipid accumulation through downregulation of CYP2E1 expression and activation of endogenous antioxidant defense systems, ultimately exhibiting potent hepatoprotective effects.

### 4.3. SP Influence on the Keap1/HO-1 Signaling Pathway and Antioxidant Defense Mechanisms

Previous research identified the Keap1/HO-1 signaling pathway as a key target for alleviating alcoholic liver damage [43,44]. In the normal state, Nrf2 is bound to Keap1 in the cytoplasm and is degraded via ubiquitination. Under oxidative stress, Keap1 undergoes conformational changes, prompting Nrf2 to translocate to the nucleus and bind to antioxidant response elements, which activate downstream antioxidant enzyme expression to protect against oxidative stress [43]. Therefore, inhibiting Keap1 activity or disrupting Keap1/Nrf2 interaction was considered an effective approach to alleviating the oxidative stress caused by alcoholic liver damage. SP nutritional intervention significantly suppressed Keap1 expression and upregulated the downstream antioxidant HO-1 protein levels. Although CA also downregulated Keap1 protein expression, it did not exhibit a significant upregulating effect on HO-1. This differed somewhat from a study by Buko et al., who reported that alcohol induction did not decrease HO-1 levels in the model group while CA treatment significantly upregulated HO-1 mRNA levels. The study also confirmed that CA activated downstream CAT and SOD expression [45]. Therefore, it was hypothesized that although CA did not exhibit a significant upregulating effect on HO-1, it still had an antioxidant impact by suppressing Keap1 protein activity and enhancing downstream antioxidant enzyme (CAT and SOD) expression. SP significantly upregulated the expression of CAT, SOD, and HO-1, an enhancement we postulate may result from synergistic interactions among the various polyphenolic compounds in SP that collectively promote HO-1 protein expression. The results indicated that SP effectively mitigated EtOH-induced oxidative stress by suppressing Keap1 protein expression and upregulating the levels of downstream antioxidant enzymes (CAT, SOD, and HO-1), consequently alleviating liver damage.

### 4.4. SP Intervention in the Inflammatory Response and Regulation of the NF-κB Signaling Pathway

Oxidative stress causes inflammation during ALD development, further exacerbating hepatic necrotic damage, and forming an inflammatory cascade with oxidative stress [12]. Previous studies have indicated that dietary polyphenols display biological activities that alleviate inflammation in the body. Do et al. reported that aqueous polyphenol extracts derived from molokhia leaves suppressed the protein expression of IL-1β, IL-6, and TNF-α, and alleviated alcohol-induced hepatic inflammation [46]. Xu et al. demonstrated that HTF downregulated the levels of IL-1β, IL-6, and TNF-α, regulated the NF-κB signaling pathway, and alleviated liver inflammation [12]. The present study showed significantly elevated IL-1β, IL-6, and TNF-α levels in the MC group, and substantial inflammatory cell infiltration in the histopathological liver sections, which was consistent with the findings of Zogona et al. [25]. SP and CA nutritional intervention significantly suppressed the IL-1β, IL-6, and TNF-α inflammatory cytokine expression, while alleviating inflammatory infiltration in the liver tissue. The SP-H group exhibited a similar ability to CA in suppressing the expression of inflammatory factors. Therefore, it was hypothesized that the other phenolics in SP played a role similar to that of CA. Previous research highlighted the anti-inflammatory activity of SP. Wang et al. reported that SP downregulated the IL-1β, IL-6, and TNF-α levels in the skin of UV-B-induced photoaging mice and alleviated the inflammatory response [22]. Wang et al. induced glycosylation injury in L929 cells using AGEs and revealed that SP inhibited the PI3K/Akt signaling pathway by suppressing the PI3K and Akt phosphorylation levels, exerting an anti-inflammatory effect [23]. The results confirmed that SP effectively reduced the expression of IL-1β, IL-6, and TNF-α, which mitigated inflammatory infiltration into liver tissues and alleviated EtOH-induced hepatic inflammatory damage in ALD mice.

The NF-κB signaling pathway directly regulates the IL-1β, IL-6, and TNF-α expression levels [1,11]. In normal physiological conditions, NF-κB binds to IκB in the cytoplasm is in an inactive state. Inducible factor stimulation activates the IKK complex, leading to IκB phosphorylation. The phosphorylated IκB undergoes ubiquitination and is subsequently subjected to proteasomal degradation, releasing NF-κB into the nucleus to regulate the expression of downstream inflammatory factors [47]. Transcription factor p65 is another key target for alleviating alcoholic liver injury [48]. Previous studies have shown that RRP and E Se tea polyphenol extracts inhibit p-p65 protein expression, thereby ameliorating the EtOH-induced hepatic inflammatory response [25,38]. This study found that chronic alcohol exposure contributed to the activation of upstream IKK and IκB, and subsequently the downstream NF-κB signaling pathway, further triggering the production of inflammatory factors and inflammatory injury. The findings of the present study indicated that SP significantly suppressed p65 phosphorylation while inhibiting IKK and IκB phosphorylation upstream of the NF-κB pathway. Furthermore, the CA nutritional intervention also downregulated the expression of p-IKK, p-IκB, and p-p65. However, compared to CA, SP-H exhibited a stronger inhibitory effect on IKK and p65 phosphorylation. These effects may be attributed to the synergistic actions of additional phenolic compounds in SP, which collectively modulate the expression of p-IKK and p-p65. SP effectively suppressed IKK, IκB, and p65 phosphorylation, modulated the NF-κB signaling pathway, downregulated IL-1β, IL-6, and TNF-α expression, and mitigated EtOH-induced liver inflammation.

### 4.5. SP Intervention in Multi-Target Modulation of the CYP2E1/Keap1/NF-κB Signaling Pathway

**Figure 8:**
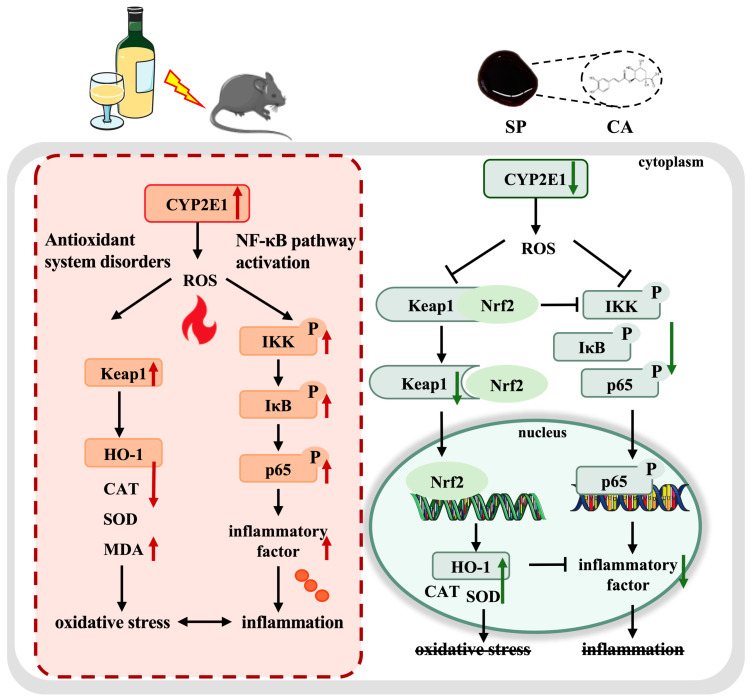
A schematic diagram indicating how SP protects ALD mice via the multi-target regulation of the CYP2E1/Keap1/NF-κB signaling pathway. Note: Red arrows represent alcohol-induced changes and green arrows represent the moderating effect of SP.

The development of ALD involves a complex interplay between multiple molecular mechanisms. Studies have demonstrated that the CYP2E1/ROS/Keap1 and NF-κB pathways are key pathways in alcohol-induced organ damage [49]. CYP2E1 is a key enzyme in alcohol metabolism, while the large amount of ROS produced during its catalysis of ethanol metabolism leads to dysregulation of the Keap1/HO-1 signaling pathway, which reduces cellular antioxidant capacity and induces oxidative stress [5,50]. Moreover, Keap1 protein overexpression also promotes IKKβ ubiquitination and degradation, triggering a cascade of NF-κB signaling pathway activation [51]. As shown in Figure 8, SP alleviated alcohol-induced liver damage by modulating the CYP2E1/Keap1/NF-κB signaling pathway at multiple levels. SP suppressed CYP2E1 expression, reduced ROS generation at its source, and mitigated the burden of oxidative stress in the liver. Moreover, SP effectively downregulated the Keap1 protein level, possibly influencing Nrf2 nuclear translocation and enhancing the expression of HO-1 and downstream antioxidant enzymes (SOD and CAT), consequently displaying a significant antioxidant impact. In addition, SP treatment effectively suppressed IKK, IκB, and p65 phosphorylation, inhibited NF-κB activation, and reduced the expression of pro-inflammatory cytokines, including IL-1β, IL-6, and TNF-α, thereby attenuating hepatic inflammatory infiltration. These results indicated that SP conferred hepatoprotective effects by inhibiting NF-κB-mediated cytokine expression and mitigating inflammation-associated liver damage. Keap1/HO-1 also modulated the inhibition of the NF-κB signaling pathway. A relatively low level of Keap1 protein expression promoted IKKβ accumulation and stabilization, consequently preventing downstream IκB and p65 activation and mitigating the hepatic inflammatory response. HO-1 directly suppressed pro-inflammatory cytokine production, while its metabolite carbon monoxide acted as a potent NF-κB inhibitor [52]. Studies confirmed that SP intervened in the cross-regulation of the CYP2E1/Keap1/NF-κB signaling pathway via multiple mechanisms. By suppressing CYP2E1 expression, SP reduced ROS production, stabilized the Keap1/HO-1 signaling pathway, and further inhibited NF-κB activation, which exerted an effective antioxidant and anti-inflammatory effect to protect against alcohol-induced liver damage.

## 5. Conclusions

This study systematically elucidated the intervention effects and molecular mechanisms of SP on alcohol-induced chronic liver damage in mice. The results demonstrated that SP significantly ameliorated alcohol-induced hepatic dysfunction in a dose-dependent manner, specifically manifested by suppressing the elevation of the liver index, reducing abnormal serum transaminase activity, and reversing lipid metabolism disorders. At the mechanistic level, SP exerted multi-target synergistic effects by effectively inhibiting CYP2E1-mediated ROS generation, modulating the Keap1 signaling pathway, and blocking NF-κB pathway activation, thereby upregulating antioxidant proteins such as CAT, SOD, and HO-1 while downregulating key inflammatory factors including IL-1β, IL-6, and TNF-α, ultimately achieving hepatoprotective effects. Notably, compared with the single-component CA, SP’s polyphenol synergistic effects demonstrated significant advantages in inhibiting hepatic TC accumulation, enhancing CAT activity, and regulating IKK and p65 phosphorylation. These findings not only revealed SP’s regulatory network through the CYP2E1/Keap1/NF-κB pathway, which blocks the oxidative stress–inflammatory cascade, but more importantly, provided a theoretical basis for developing natural hepatoprotective functional foods based on agricultural by-products. Importantly, this study provides the first experimental evidence supporting SP’s potential as a dietary supplement for preventing alcohol-induced liver damage, offering new possibilities for the high-value utilization of natural active components from agricultural byproducts. However, the specific contribution ratios of individual polyphenol components in SP remain to be elucidated, and their synergistic mechanisms require further clarification through component knockout or combinatorial synthesis.

## Data Availability

Data will be made available on request.
